# Constitutive and insect‐induced transcriptomes of weevil‐resistant and susceptible Sitka spruce

**DOI:** 10.1002/pei3.10053

**Published:** 2021-06-09

**Authors:** Justin G. A. Whitehill, Macaire M. S. Yuen, Jörg Bohlmann

**Affiliations:** ^1^ Michael Smith Laboratories University of British Columbia Vancouver BC Canada; ^2^ Department of Forestry and Environmental Resources North Carolina State University Raleigh NC USA; ^3^ Department of Botany University of British Columbia Vancouver BC Canada; ^4^ Department of Forest and Conservation Sciences University of British Columbia Vancouver BC Canada

**Keywords:** conifer genomics, defense syndrome, forest health, microbiome, plant defense, plant insect interaction, plant resistance, RNA‐seq factorial design

## Abstract

Spruce weevil (*Pissodes strobi*) is a significant pest of regenerating spruce (*Picea*) and pine (*Pinus*) forests in North America. Weevil larvae feed in the bark, phloem, cambium, and outer xylem of apical shoots, causing stunted growth or mortality of young trees. We identified and characterized constitutive and weevil‐induced patterns of Sitka spruce (*Picea sitchensis*) transcriptomes in weevil‐resistant (R) and susceptible (S) trees using RNA sequencing (RNA‐seq) and differential expression (DE) analyses. We developed a statistical model for the analysis of RNA‐seq data from treatment experiments with a 2 × 3 factorial design to differentiate insect‐induced responses from the effects of mechanical damage. Across the different comparisons, we identified two major transcriptome contrasts: A large set of genes that was constitutively DE between R and S trees, and another set of genes that was DE in weevil‐induced S‐trees. The constitutive transcriptome unique to R trees appeared to be attuned to defense, while the constitutive transcriptome unique to S trees was enriched for growth‐related transcripts. Notably, a set of transcripts annotated as “fungal” was detected consistently in the transcriptomes. Fungal transcripts were identified as DE in the comparison of R and S trees and in the weevil‐affected DE transcriptome of S trees, suggesting a potential microbiome role in this conifer‐insect interaction.

## Graphical abstract

Overview of differentially expressed (DE) genes. Number of DE genes in R and S trees. Only two major comparisons contained DE contigs and are defined as the constitutive difference (blue circle) between R and S trees, and genes DE following weevil activity in S trees defined as weevil induced (orange circle).

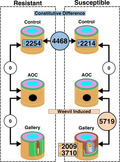

## 1 INTRODUCTION

Spruce weevil (*Pissodes strobi* Peck.; a.k.a. white pine weevil) is a significant pest of regenerating spruce forests in North America (Ebata, 1991). Repeated damage from weevil larvae results in mostly, growth deformations and therefore poor lumber quality, stunted growth (shrubby appearance), or, more rarely, tree death (Gara & Wood, 1989). The life cycle of the weevil and its interaction with the host tree involves two major phases, previously defined as the exophase and endophase (Whitehill & Bohlmann, 2019). In brief, during the exophase, adult weevils live outside of the tree, feed on the bark without causing substantial damage, until females deposit eggs in oviposition holes at the tip of the previous year apical shoot (PYAS). In the endophase, inside of the tree, hatched larvae feed basipetally on the bark, phloem, cambium, and outer xylem of the PYAS, which disrupts the flow of water and nutrients and destroys apical growth. The endophase is the most destructive life stage of the weevil until it reaches pupation and eventually emergence of young adults. Young trees between 2 and 20 years of age are particularly vulnerable to the weevil, but this range can substantially vary depending on factors such as the level of weevil pressure and rate of tree growth.

The introgression of weevil resistant (R) spruce genotypes into forest landscapes is currently the primary means for tree breeding programs to mitigate weevil damage (King & Alfaro, 2009; King et al., 2011; Kiss & Yanchuk, 1991; Whitehill & Bohlmann, 2019). In the Pacific Northwest, Sitka spruce (*P*. *sitchensis*) is particularly susceptible to this insect (King & Alfaro, 2009). However, weevil‐resistant Sitka spruce genotypes have been discovered. Durable resistance involves a synergistic defense syndrome of at least two defense traits: abundance of cortical stone cells and terpene‐rich oleoresin (Krokene, 2016; Whitehill & Bohlmann, 2019; Whitehill, Henderson, Schuetz, et al., 2016; Whitehill, Henderson, Strong, et al., 2016; Whitehill et al., 2019). While these two traits appear to provide much of the effective defense against weevil attack, additional factors such as early induced responses may contribute to the outcome of spruce‐weevil interactions (Robert & Bohlmann, 2010; Whitehill & Bohlmann, 2019).

As part of unveiling the genomic portrait of spruce‐weevil interactions, we have used RNA‐seq to capture the host transcriptome component. Analysis of RNA‐seq data in plant‐insect interactions often focuses on differentially expressed (DE) genes and may apply complex factorial experimental designs that target unique aspects of the ecological interaction. For spruce‐insect systems, various treatments have been used to differentiate host responses to artificial wounding from molecular signatures affected by real insect attack (Byun‐McKay et al., 2003, 2006; Franceschi et al., 2002; Mageroy, Christiansen, et al., 2020; Mageroy, Wilkinson, et al., 2020; Martin et al., 2002; Ralph et al., 2006, 2008; Schiebe et al., 2012; Whitehill et al., 2019). Genes that respond to mechanical wounding can be removed from DE analyses with the goal to identify insect‐specific responses. Statistical models used for transcriptome analyses often rely on open‐source software packages in R. For example, R‐based approaches were used for the DE analysis in RNA‐seq datasets to explore the molecular underpinnings of methyl jasmonate‐induced resistance against bark beetles in Norway spruce (*P*. *abies*; Mageroy, Wilkinson, et al., 2020).

Here, we expanded the analyses of a previously described RNA‐seq resource for Sitka spruce‐weevil interactions (Whitehill et al., 2019). Specifically, in this study, we provide a detailed analysis of insect‐specific transcriptome responses and include microbial signatures in our overall analysis. The results from this analysis highlighted two biologically relevant contrasts: genes that are DE between resistant (R) and susceptible (S) trees and genes that are DE between S trees challenged by weevil larvae relative to mechanical wounding or untreated controls. The DE genes identified in the first contrast were defined as the “constitutive difference” (CD); the DE genes from the second contrast were defined as “weevil‐induced” (WI). In previous work, signatures of fungi in spruce RNA‐seq datasets were removed from DE analyses (Delhomme et al., 2014, 2015; Whitehill et al., 2019). This is despite fungal transcripts being consistently detected in replicated RNA‐seq libraries (Whitehill et al., 2019). In the transcriptome analysis described here, we purposely kept genes annotated as non‐plant, as they may provide clues towards a role of a microbiome associated with the host tree or with the insect in the spruce‐weevil interaction.

## 2 MATERIALS AND METHODS

### 2.1 Plant materials

Origins and growing conditions of clonally propagated R and S Sitka spruce genotypes were described previously (Robert & Bohlmann, 2010; Whitehill, Henderson, Schuetz, et al., 2016; Whitehill, Henderson, Strong, et al., 2016; Whitehill et al., 2019). In brief, the R genotype H898 derives from a highly weevil resistant population of Sitka spruce located in the low elevation mainland of British Columbia, Canada (49°14′N; 122°36′W). The S genotype Q903 is from the Haida Gwaii Islands (53°55′N; 132°05′W). For experiments we used 5‐year‐old grafted saplings of R and S trees produced in 2008. At time of experiments trees were ~1.5 m in height. Plants were maintained year‐round in a nursery located outside on the University of British Columbia Vancouver campus (49°15′40.82″N; 123°15′09.89″W), British Columbia, Canada.

### 2.2 Experimental design

The experimental design was previously described in Whitehill et al. (2019). Here, we present the experimental procedures for downstream analyses used in the present study (Figure S1). Twelve R and twelve S trees were used for RNA‐seq experiments with four trees (biological replicates) used for each genotype × treatment combination. Treatments started on May 20th, 2013 (465 GDD) and concluded July 2nd, 2013 (923 GDD). Trees were arranged in a completely randomized design. One of three treatments were applied within a section of 0–2 cm from the tip of the PYAS to mimic the site of natural oviposition by weevils in the field (Whitehill, Henderson, Schuetz, et al., 2016). The average PYAS length where treatments were applied was 26.6 ± 2.2 cm (R) and 34.4 ± 2.0 cm (S). Needles within this section were removed with a razor blade to facilitate treatment applications. The three treatments were (1) untreated control, (2) artificial oviposition chambers (AOC), and (3) galleries, which were produced by placing individual weevil eggs into AOCs and letting larvae develop. Experiments were performed with four biological replicates for each genotype (R, S) × treatment [(1), (2), (3)]. For treatments (2) and (3), a Dremel^®^ with 1 mm drill bit was used to produce 20 AOCs per tree. AOCs were placed below the base of needles in the middle of sterigmatal ridges into the outer bark to emulate natural oviposition sites as detailed in Whitehill et al. (2019). For (3), 10 AOCs with no sign of resin flooding were inoculated with a single weevil egg per chamber. Weevil eggs were isolated from apical shoots of naturally attacked trees at the Kalamalka Research Station in Vernon, British Columbia, Canada (50°16′00″N; 119°16′18″W). Egg isolations (Whitehill, Henderson, Strong, et al., 2016) and collection conditions (Whitehill et al., 2019) were described previously. The top 2 cm of the PYASs were wrapped with parafilm to protect treatment sites. Trees were kept under natural conditions outside at the University of British Columbia Vancouver Point Grey campus for the duration of the experiment. PYAS samples were collected as illustrated in Figure S1 at the time when weevil larvae entered the final stage of development in S trees. The experiment was performed over a 50‐day period (Whitehill et al., 2019).

### 2.3 Collection of bark samples

PYASs were harvested and bark samples, which consisted of outer bark, cortex, phloem, cambium, and traces of outer xylem, isolated on July 2nd at 923 growing degree‐days (GDD). Details regarding developmental stage of weevil larvae were previously described (Whitehill et al., 2019). Briefly, R tree larvae were in the 3rd and S tree larvae were in the 4th instar of development at the time of sample collection. Samples for control treatments (1) and AOC treatments (2) were harvested at the top 0–2 cm of the PYAS; samples for gallery treatments (3) were collected by excising the bark surrounding the final weevil‐feeding site as described in Whitehill et al. (2019; Figure S1). Weevil gallery tissues used for RNA‐seq included the lower 2 cm of a larvae gallery with a 1 cm border of adjacent bark tissue including the larval feeding margin after removal of the insect and fecal materials. Gallery tissues from a given individual tree were pooled and treated as a single biological replicate. All samples were flash frozen in liquid N_2_ and stored at −80℃ until RNA extraction.

### 2.4 Transcriptome resource and DE analysis

The complete methods for RNA extraction, Illumina HiSeq library preparation, sequencing on the HiSeq2000 platform, raw data processing, quality assessments, and de novo transcriptome assembly were previously described (Whitehill et al., 2019); except, for the present analyses, contigs with annotations to fungal, insect, and other (bacterial, virus, etc.) origins were not removed from the final assembly. Raw reads are available at NCBI SRA (BioProject PRJNA398042). DE analyses were performed using the voom/limma package (Law et al., 2014) in R on coding sequences (CDS), predicted by TransDecoder (Haas et al., 2013), with expression of at least 1 count per million (cpm) in at least two libraries and quantified using Sailfish (version 0.10.0) with default parameters (Patro et al., 2014). Statistically significant (adjusted *p* ≤ .05) CDS with a | log_2_ fold change | ≥2 were annotated with BLASTP against the UniProt database with an e‐value cutoff at 1e^−20^ (Tables S1 and S2). Gene ontology annotations were extracted from corresponding best hit UniProt ID. Taxonomic lineage information of the best hit was extracted from the NCBI Taxonomy Database. Complete GO term annotations and taxonomic lineage are available in Table S2.

### 2.5 RNA‐seq gene expression model for a 2 × 3 factorial design

To remove the effect of wounding on contigs identified as DE in gallery treatments (3), analyses were modeled as a 2 × 3 factorial design for two genotypes and three treatment combinations. A full analysis using the 2 × 3 factorial model revealed only two comparisons had large differences in gene expression, one being the comparison between control treatments (1) of resistant (R) and susceptible (S) trees defined as constitutive differences (CD) and the other being DE genes between AOC (2) and gallery (3) treatments defined as weevil‐induced (WI) in S trees. A complete description of the model and analysis is available at https://github.com/myuen/White_Pine_Weevil_DE. DE transcripts were identified as WI using the interactive model by removing the AOC wounding effect (2) from the compound interactive effect of the gallery treatment (3) and are assumed to represent genes affected specifically by weevil activity.

### 2.6 Comparisons of DE transcriptome contrasts

Comparisons of statistically significant (adjusted *p* ≤ .05 and |log2 fold change| ≥2) DE contigs from the two main contrasting comparisons were performed. Genes DE in the CD contrast were compared against the WI contrast. Genes were grouped by expression patterns into eight different sets (Table S2). Genes with higher constitutive expression exclusively in CD R trees were labeled Set 1, while genes with lower expression exclusively in CD S trees were labeled Set 2. WI genes exclusively up‐regulated or down‐regulated in S trees were labeled Sets 3 and 4, respectively. Four additional contrasts were found at the intersection of the two‐major CD versus WI contrasts. These were defined as higher CD expression in R trees and WI up‐regulated in S trees (Set 5), higher CD expression in S trees and WI up‐regulated in S trees (Set 6), higher CD expression in R trees and WI down‐regulated in S trees (Set 7), and higher CD expression in S trees and lower WI expression in S trees (Set 8). Heatmaps were generated for DE contigs in Sets 5–8 using the D3HEATMAP package (Cheng, 2016) from R (R Core Team, 2013). Volcano plots of overall DE patterns among comparison groups were generated using ggplot2. GO term enrichment analyses were performed with the R package “topGO” version 2.38.1 (Alexa & Rahnenfuhrer, 2019). GO terms enriched in Sets 1–4 were identified using Fisher's exact test statistic (*a* ≤ 0.01) with a default graph method “weight01” and node size of 10. Codes for DE analyses and generation of figures are available on GitHub (https://github.com/myuen/Intersection_analysis).

## 3 RESULTS

### 3.1 Overall patterns of DE contigs

RNA‐seq data were generated from 24 cDNA libraries comprising PYAS bark tissue from R and S trees under three different treatment conditions, control (1), AOC (2), and gallery (3). The overall patterns of gene expression between genotype and treatment groups are shown in Figure S2. In the control transcriptomes, 4468 genes were DE between R and S trees. Of these genes, 2254 had higher transcript levels in the R genotype and 2214 had higher transcript levels in the S genotype (Table 1). The genes of this contrast were defined as “constitutive difference” (CD) between R and S trees and annotated using UniProt terms (3731 genes—Table S2) or GO terms (942 genes—Table S2). In the “weevil‐induced” (WI) transcriptomes, we found 5719 genes were DE between gallery samples and AOC samples in S trees. Of the genes of the WI contrast, 2009 were up‐regulated and 3710 were down‐regulated in response to the prolonged activity of developing weevil larvae (Figure S2). No genes were found DE as a result of prolonged weevil activity in R trees. It is important to note that the experiment was designed to capture transcriptome signatures in prolonged treatments of 50 days that resemble the mature stage of a natural weevil infestation. This design may not have captured the early and transiently induced response in R and S trees. Early induced responses have been the topic of previous work (Lippert et al., 2007; Ralph et al., 2006). Effects of AOC alone were removed using a 2 × 3 factorial model to identify DE responses likely to be specific to weevil activity. WI genes were annotated using UniProt terms (3710 genes) or GO terms (1407 genes; Table S2).

**TABLE 1:** Total number of differentially expressed (DE) genes constitutively different (CD) between R and S trees or weevil induced (WI) in S trees that received a BLASTP and GO term annotation. Contigs with an adjusted *p* ≤ .05 and |log‐2 *Fold* *Change|* ≥2 were considered DE

	Constitutive difference	Weevil induced
Number of DE genes (≥2‐fold expression; adj. *p* < .05)	4,468	5,719
^a^Higher expression in R or ^b^Up‐regulated in S	2,254^a^	2,009^b^
^c^Higher expression in S or ^d^Down‐regulated in SG	2,214^c^	3,710^d^
Number of DE genes with UniProt BLASTP Annotations	3,731	4,913
Number of DE genes with GO annotations (Biological Process)	942	1,407

The superscript letters indicate which of the numbers are higher expressed or upregulated and correspond to the text in the same row.

### 3.2 Comparison of constitutive different (CD) and weevil induced (WI) transcriptomes

**FIGURE 1:**
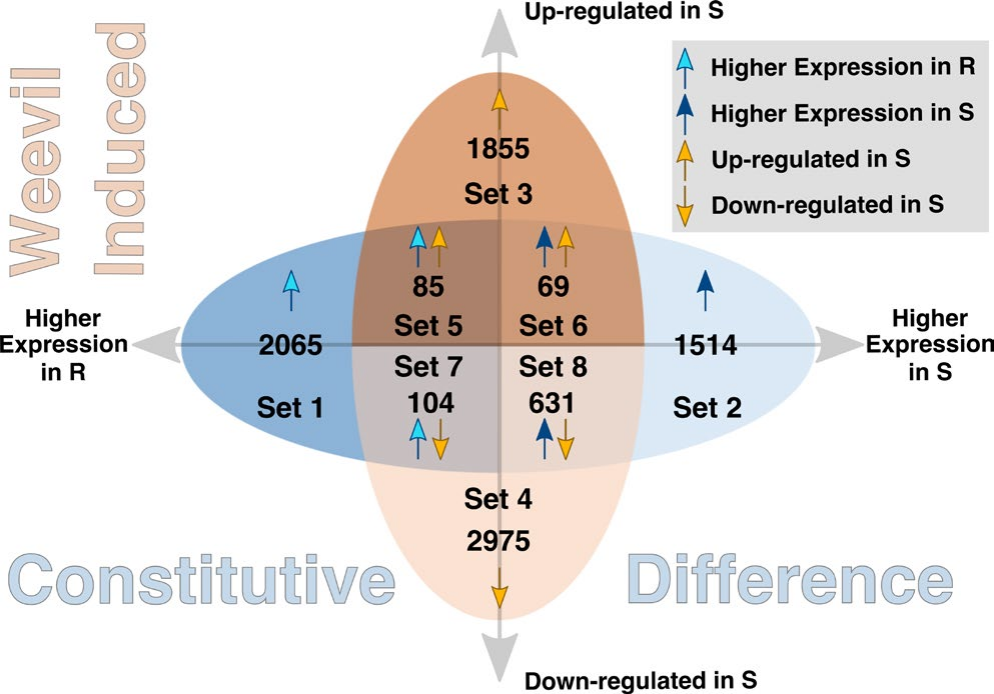
Venn diagram of CD and WI genes. Venn diagram separates DE CD (blue colors) and WI (orange colors) into eight Sets. CD genes are shown along the *x*‐axis and WI genes along the *y*‐axis

We compared the two contrasts to identify unique and shared genes between the CD (Figure 1; *x*‐axis) and WI (*y*‐axis) transcriptomes. We found eight sets of genes that differed by expression patterns, including 3579 genes unique to the CD contrast (Sets 1 and 2), 4830 genes unique to the WI contrast (Sets 3 and 4), and 889 genes shared between the CD and WI contrasts (Sets 5–8).

#### 3.2.1 Genes unique to the CD contrast

**FIGURE 2:**
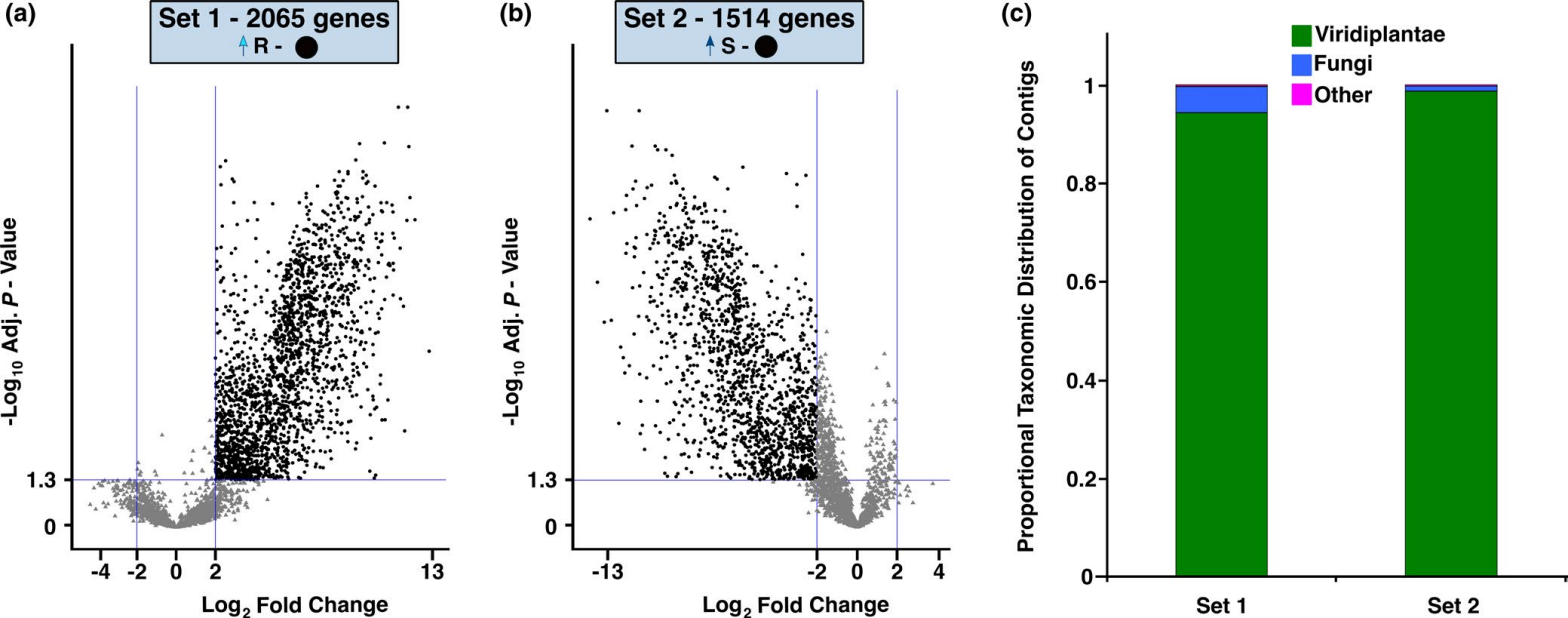
Genes unique to the CD contrast. Volcano plots for DE CD genes with higher expression in: (a) R (Set 1—●) trees and (b) S (Set 2—●) trees. DE CD genes are compared with the same genes from the WI contrast (▲). (c) Taxonomic annotation of Set 1 and 2 genes. The distribution of taxonomic groups from Set 1 and Set 2 was normalized to the total number of DE genes within a Set and grouped into three categories that include viridaeplantae, fungi, and others

Sets 1 and 2 consist of genes with constitutively higher levels of expression in R trees and S trees, respectively. Volcano plots of fold change values plotted against the negative log of *p*‐values for all DE genes show the expression of these genes in the CD (Figure 2a) and in WI (Figure 2b). It shows a clear separation between significantly (adjusted *p* ≤ .05 with a | log_2_ fold change | ≥2) DE genes of Sets 1 and 2 (Figure 2a,b). The majority of Set 1 and 2 genes were annotated as viridiplantae (1633 genes in Set 1; 1266 in Set 2), and smaller numbers of genes were annotated as fungi (93 Set 1; 14 Set 2) or other (6 Set 1; 3 Set 2; Figure 2c). Notably, DE genes annotated as fungi were more abundant in R trees (5.7% of annotated genes in Set 1) than in S trees (1.1% of annotated genes in Set 2).

To further explore potential biological functions of DE genes in Sets 1 and 2, Gene Ontology (GO) biological process terms were evaluated. A majority of genes fell into six GO term categories and accounted for 38.2% and 42.7% of all annotated genes with assigned GO terms for Set 1 (R) and Set 2 (S), respectively. The six GO term categories include signal transduction (21.9% Set 1, 26.7% Set 2), lipid metabolic process (5.2% Set 1, 3.8% Set 2), regulation of transcription—DNA template (3.5% Set 1, 4.6% Set 2), carbohydrate metabolic process (3.7% Set 1, 2.4% Set 2), pectin catabolic process (3.1% Set 1, 1.7% Set 2), and translation (0.8% subset 1, 3.4% subset 2). S tree CD transcriptomes were enriched for 126 GO terms; R tree CD transcriptomes were enriched for 123 GO terms (Table S2). We focused on GO terms that fell into two main categories, growth and defense and evaluated differences between R and S trees. We defined growth categories by terms containing the keywords “growth”, “seed”, “embryo”, “chloro‐”, “photo‐”, and “reproduction”. Defense categories were defined by keywords “defense”, “response”, “secondary metabol‐”, “resistance”, “terpenoid”, “isoprenoid”, and “virus induced gene silencing”. For CD genes with higher expression in R trees (Set 1), ten of the “defense” GO terms and six of the “growth” terms represented 5% and 1.7%, respectively, of all annotated genes. For CD genes with higher expression in S trees (Set 2), nine of the “defense” terms and thirteen “growth” terms were identified representing 2.9% and 6.2%, respectively. Fisher's exact test was used to evaluate statistically significant enriched GO terms within Set 1 and Set 2. Two terms were enriched in Set 1 and another two terms in Set 2. The growth term, photosynthesis light reaction, was significantly enriched in the S tree dataset (Table 2). Overall, these results suggested that the constitutive transcriptome signature of R trees is more attuned to “defense”, while the constitutive transcriptome signature of S trees is more enriched towards ‘growth’.

**TABLE 2:** Fisher's exact enrichment analysis of GO term annotations of DE genes between sets. Statistically significant (*α* = 0.01) comparisons of differences in gene ontology functions for DE genes between Set 1 versus Set 2 and Set 3 versus Set 4

GO term ID	GO term	*p*‐value	Comparison	Significant in set #
GO:0051726	Regulation of cell cycle	<.001	Set 1 versus Set 2	1
GO:0042737	Drug catabolic process	<.001	Set 1 versus Set 2	1
GO:0055114	Oxidation‐reduction process	<.001	Set 1 versus Set 2	2
GO:0019684	Photosynthesis, light reaction	<.001	Set 1 versus Set 2	2
GO:0005975	Carbohydrate metabolic process	<.001	Set 3 versus Set 4	3
GO:0009058	Biosynthetic process	<.001	Set 3 versus Set 4	3
GO:0016998	Cell wall macromolecule catabolic process	<.001	Set 3 versus Set 4	3
GO:0006032	Chitin catabolic process	<.001	Set 3 versus Set 4	3
GO:1901605	Alpha‐amino acid metabolic process	<.001	Set 3 versus Set 4	3
GO:1901362	Organic cyclic compound biosynthetic process	<.001	Set 3 versus Set 4	3
GO:0009108	Coenzyme biosynthetic process	<.001	Set 3 versus Set 4	3
GO:0019438	Aromatic compound biosynthetic process	<.001	Set 3 versus Set 4	3
GO:0017144	Drug metabolic process	<.001	Set 3 versus Set 4	3
GO:0009072	Aromatic amino acid family metabolic process	<.001	Set 3 versus Set 4	3
GO:0042737	Drug catabolic process	<.001	Set 3 versus Set 4	3
GO:0032787	Monocarboxylic acid metabolic process	<.001	Set 3 versus Set 4	3
GO:0006790	Sulfur compound metabolic process	<.001	Set 3 versus Set 4	3
GO:0018130	Heterocycle biosynthetic process	<.001	Set 3 versus Set 4	3
GO:0031408	Oxylipin biosynthetic process	<.001	Set 3 versus Set 4	3
GO:0072330	Monocarboxylic acid biosynthetic process	.005	Set 3 versus Set 4	3
GO:0007018	Microtubule‐based movement	<.001	Set 3 versus Set 4	4
GO:0030001	Metal ion transport	<.001	Set 3 versus Set 4	4
GO:0042546	Cell wall biogenesis	<.001	Set 3 versus Set 4	4
GO:0016042	Lipid catabolic process	<.001	Set 3 versus Set 4	4
GO:0006281	DNA repair	<.001	Set 3 versus Set 4	4
GO:0051173	Positive regulation of nitrogen compound metabolic process	<.001	Set 3 versus Set 4	4
GO:0010604	Positive regulation of Macromolecule metabolic process	<.001	Set 3 versus Set 4	4
GO:0031325	Positive regulation of cellular metabolic process	<.001	Set 3 versus Set 4	4
GO:1903047	Mitotic cell cycle process	.002	Set 3 versus Set 4	4
GO:0009734	Auxin‐activated signaling pathway	.002	Set 3 versus Set 4	4
GO:0006260	DNA replication	.003	Set 3 versus Set 4	4
GO:0033043	Regulation of organelle organization	.003	Set 3 versus Set 4	4
GO:0000226	Microtubule cytoskeleton organization	.003	Set 3 versus Set 4	4
GO:0007165	Signal transduction	.004	Set 3 versus Set 4	4
GO:0019953	Sexual reproduction	.005	Set 3 versus Set 4	4
GO:0006355	Regulation of transcription, DNA‐templated	.005	Set 3 versus Set 4	4
GO:0010564	Regulation of cell cycle process	.007	Set 3 versus Set 4	4
GO:0030245	Cellulose catabolic process	.008	Set 3 versus Set 4	4
GO:0071103	DNA conformation change	.008	Set 3 versus Set 4	4
GO:0045859	Regulation of protein kinase activity	.008	Set 3 versus Set 4	4
GO:0048523	Negative regulation of cellular process	.008	Set 3 versus Set 4	4

#### 3.2.2 Genes unique to the WI contrast

**FIGURE 3:**
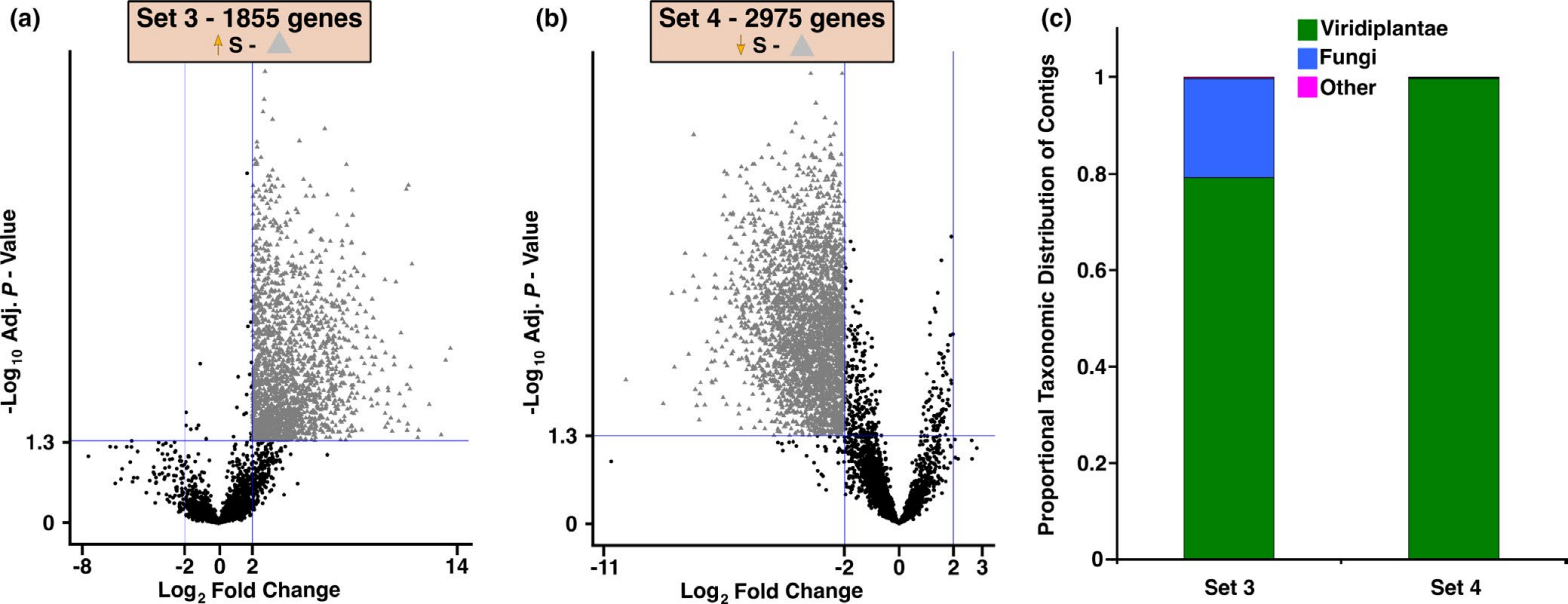
Genes unique to the WI contrast. Volcano plots for DE WI genes that are: (a) up‐ (Set 3—▲ right quadrant) and (b) down‐regulated (Set 4—▲ left quadrant) in S trees are compared with the same genes from the CD contrast (●). (c) Taxonomic annotation of Set 3 and 4 genes. The distribution of taxonomic groups was normalized to the total number of DE genes within a subset and grouped into three categories that include viridaeplantae, fungi, and other

Set 3 and Set 4 consist of DE WI genes up‐regulated or down‐regulated, respectively, in bark tissue surrounding weevil galleries of S trees (Figure 1). Volcano plots were used to visualize the log_2_‐fold change of DE WI genes compared against the negative log *P*‐values for the same transcripts in control samples to show the separation of DE genes between Set 3 and Set 4 (Figure 3a,b). The majority of Set 3 and Set 4 genes were annotated as viridiplantae (1329 genes in Set 3; 2,513 in Set 4; Figure 3c). A relatively large number of genes (345; 20.6% of annotated genes) were annotated as fungi in Set 3, while only 0.2% (5 contigs) of all genes were identified as fungi in Set 4. Fungal transcripts in galleries of S trees (Set 3) may originate from fungi introduced with weevil inoculation and proliferating with gallery formation, or they may represent endophytes favored by the presence of weevil larvae. The same fungal transcripts were present constitutively in R trees, but were not DE, and are shown in the volcano plot as black circles (Figure 3a,b). Other taxonomic annotations represented by genes of unknown origin were low in abundance (5 in Set 3; 1 in Set 4).

Sets 3 and Set 4 were comprised of genes that fell into 326 GO term categories. 105 terms were more represented in Set 3 and 221 terms more abundant in Set 4 (Table S2). The most abundant GO term annotation in Set 3 was “carbohydrate metabolic process” (14.6%) while “biosynthetic process” (5.1%) was the most abundant term in Set 4. WI genes up‐regulated in Set 3 consisted of eight “defense” and two “growth” GO terms representing 10% and 0.6%, respectively, of all annotated genes. WI genes down‐regulated in Set 4 contained 15 “defense” and nine “growth” terms representing 4% and 2%, respectively. A Fisher's exact test revealed 16 (Set 3) and 21 (Set 4) GO term annotations were significantly enriched. Notably, the term “chitin catabolic process” was enriched in Set 3, consistent with observations of fungal transcripts in this set. The GO terms “aromatic compound biosynthetic process” and “oxylipin biosynthetic process”, which are relevant to plant defense, were significantly enriched in Set 3. Conversely, GO terms enriched and down‐regulated in Set 4 include processes related to cellular damage (i.e., DNA repair, negative regulation of cellular process) and growth (i.e., sexual reproduction, auxin‐activated signaling pathway; Table 2).

#### 3.2.3 Genes shared between CD and WI contrasts

Genes of Sets 5–8 were shared between the CD and WI transcriptomes, with four different expression patterns (Figure 1):

Set 5: CD higher expressed in R trees and WI up‐regulated in S trees.

Set 6: CD higher expressed in S trees and WI up‐regulated in S trees.

Set 7: CD higher expressed in R trees and WI down‐regulated in S trees.

Set 8: CD higher expressed in S trees and WI down‐regulated in S trees.

**FIGURE 4:**
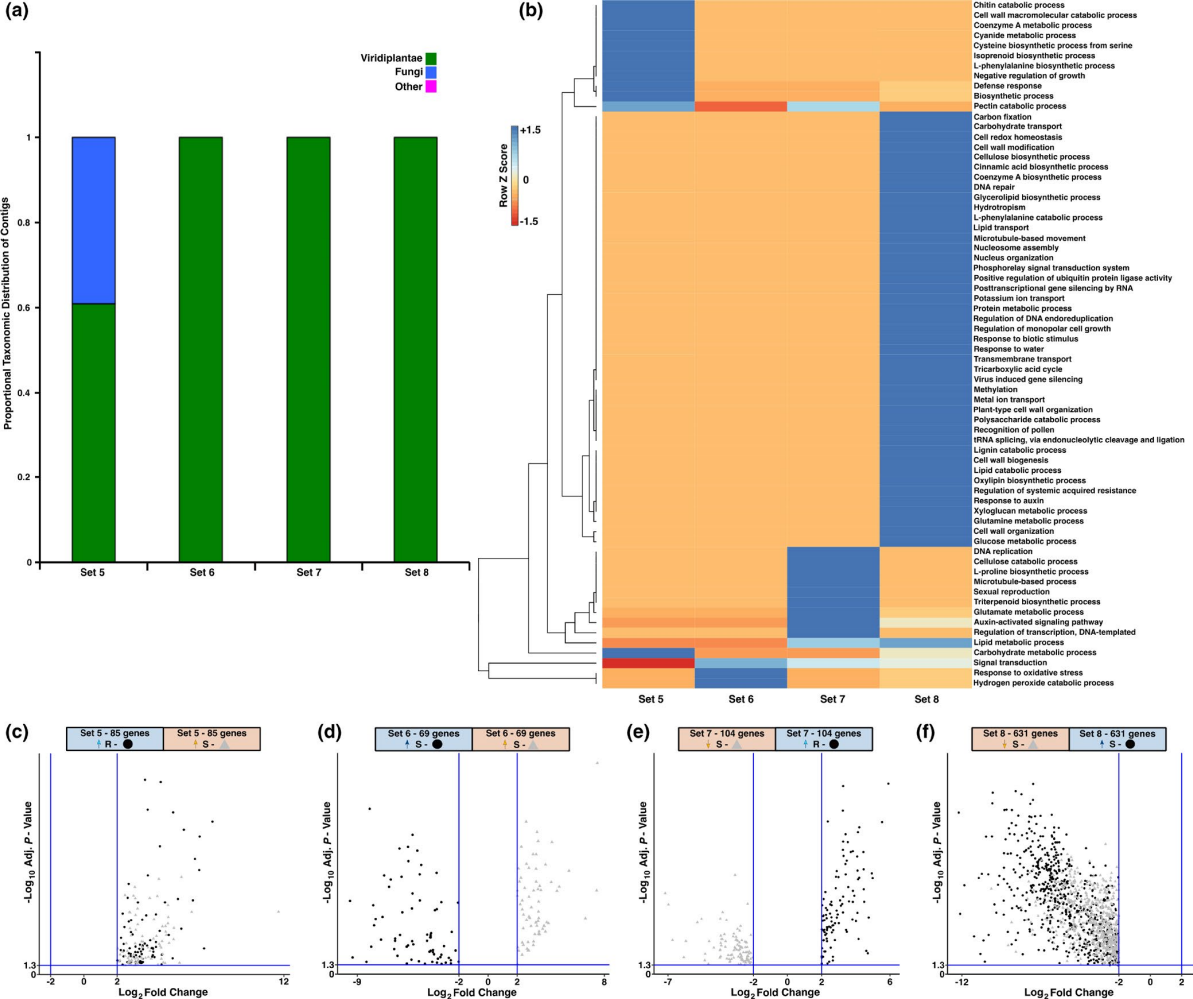
Intersection of CD and WI genes. (a) Taxonomic annotation of Set 5–8 genes. The distribution of taxonomic groups was normalized to the total number of DE genes within a subset and grouped into three categories that include viridaeplantae, fungi, and other. (b) Hierarchical clustering analysis (heatmap) of Set 5–8 genes with assigned GO term annotations. GO term categories are normalized to the total number of genes that received a GO annotation. Dark blue indicates a majority proportion of GO term representatives are found within a particular set while red indicates no representatives were present. Intermediate lighter blue and red colors indicate at least 1 representative is present. Volcano plots of DE contigs are presented in: (c) Set 5—CD higher expressed in R trees ● and WI up‐regulated in S trees ▲; (d) Set 6—CD higher expressed in S trees ● and WI up‐regulated in S trees ▲; (e) Set 7—CD higher expressed in R trees ● and WI down‐regulated in S trees ▲; and (f) Set 8—CD higher expressed in S trees ● and WI down‐regulated in S trees ▲

Genes of Sets 5, 6 and 7 may contribute to weevil defense or resistance. Volcano plots show expression patterns of DE transcripts Sets 5–8 (Figure 4c–f). The majority of genes were annotated as viridiplantae (Figure 4a). However, 39% of annotated genes in Set 5 were identified as fungal, while fungal genes were not detected in Sets 6, 7 and 8. This is noteworthy because Set 5 comprises genes that were constitutively more highly expressed in R trees and were up‐regulated in S trees in response to long‐term weevil activity and may be of particular interest for defense.

A heatmap of the normalized GO biological process terms for Sets 5–8 revealed distinct differences (Figure 4b). In Set 5, the most abundant GO term was “carbohydrate metabolic process” (15% of annotated transcripts). Only 3 GO annotations were available for Set 6 and include “signal transduction”, “response to oxidative stress”, and “hydrogen peroxide catabolic process”. In Set 7 and Set 8, genes annotated as “signal transduction” (25% and 23%, respectively, of annotated transcripts) were the most abundant GO term.

### 3.3 DE fungal genes in CD and WI datasets

**FIGURE 5:**
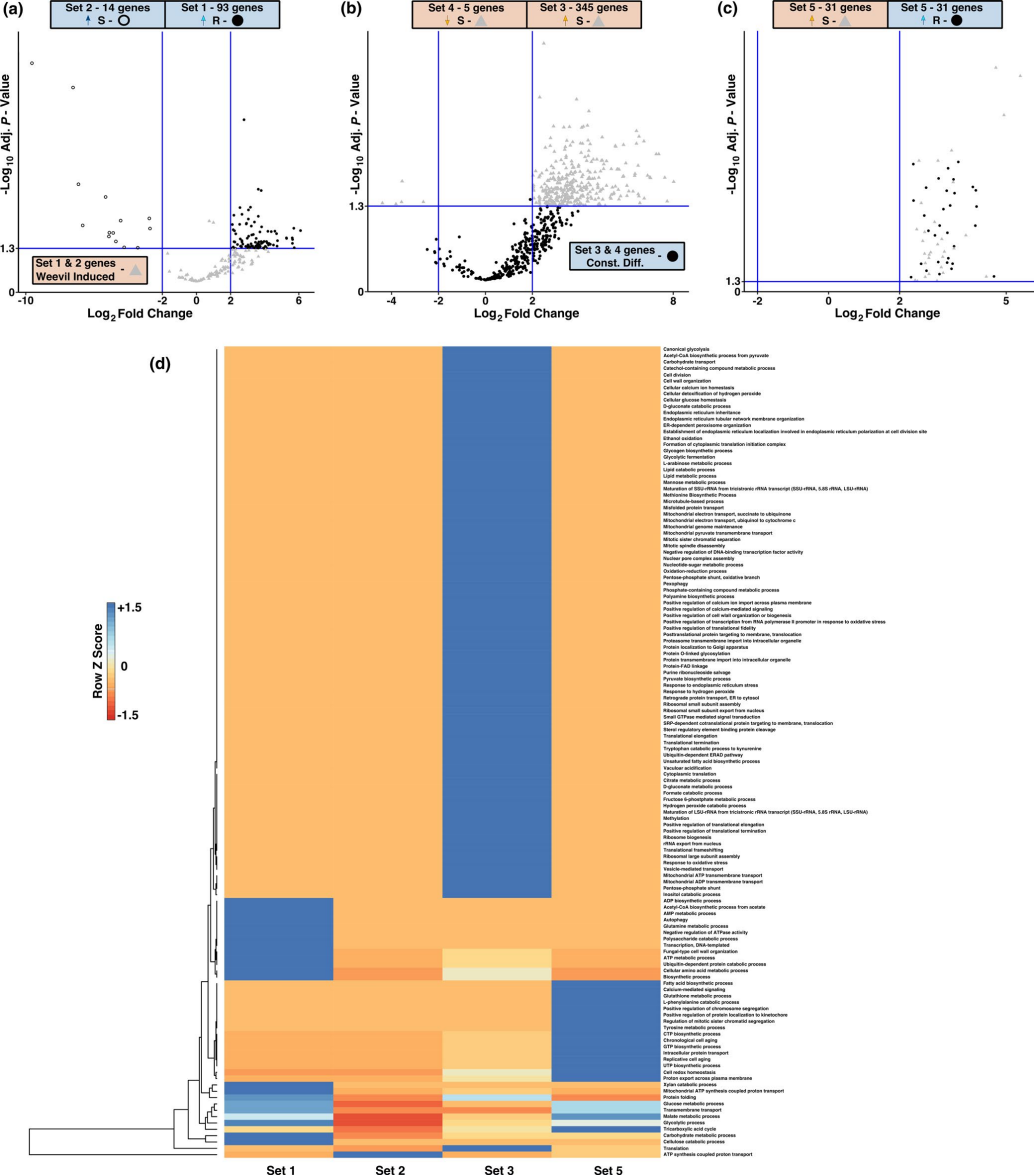
DE fungal genes in the CD and WI datasets. (a) Volcano plots for fungal genes DE in Set 1 (● right quadrant) and Set 2 (○ left quadrant) compared with the same genes from the WI (▲) contrast. (b) Volcano plots for fungal genes DE in Set 3 (▲ right quadrant) and Set 4 (▲ left quadrant) compared with the same genes from the CD (●) contrast. (c) Volcano plot of fungal genes DE in Set 5. Fungal genes from Set 5 are DE in the CD (higher expression in RC ●) and WI (up‐regulated in SG ▲) contrast. (d) Hierarchical clustering analysis (heatmap) of Sets 1, 2, 3 and 5 DE contigs with an assigned GO term annotation. Set 4 fungal genes were not included as no DE contig (5 total) received a GO term annotation. GO term categories are normalized to the total number of genes that received a GO annotation. Dark blue indicates a majority proportion of GO term representatives are found within a particular set while red indicates no representatives were present. Intermediate lighter blue and red colors indicate at least 1 representative is present

Fungal genes were consistently detected in replicated transcriptomes and in different treatments of R and S trees. DE fungal genes were present in Sets 1–5. Volcano plots of DE fungal genes showed differences in expression patterns between Sets (Figure 5a–c). A heatmap of normalized GO terms for biological process annotations for fungal transcripts was used to visualized patterns among sets. (Figure 5d).

## 4 DISCUSSION

Tree breeding for insect resistance stands to benefit from the application of genomics‐based tools, such as genetic markers identified from genome and transcriptome analyses or genomic selection to overcome challenges associated with screening for pest resistance (Beaulieu et al., 2020; Isik, 2014; Lenz et al., 2019; Neale & Kremer, 2011; Plomion et al., 2011). Previous comparisons of R and S trees elucidated defense mechanisms that contribute to weevil resistance, such as oleoresin terpenes and stone cells (Hall et al., 2011; Roach et al., 2014; Robert & Bohlmann, 2010; Robert et al., 2010; Whitehill & Bohlmann, 2019; Whitehill, Henderson, Schuetz, et al., 2016; Whitehill, Henderson, Strong, et al., 2016; Whitehill et al., 2019). The present transcriptome analysis was performed using a statistical model to identify weevil specific responses and assess differences in gene expression between R and S trees and treatments. Under the conditions of long‐term weevil exposure, we did not detect weevil‐induced DE genes in R trees at 50 days after mechanical wounding or onset of weevil exposure. While these results may suggest that resistance in the R genotype involves primarily constitutive defenses as opposed to induced defenses, it is important to note that rapidly‐induced and transient defense gene responses would have been missed in this experimental design. Conversely, S spruce showed an induced response at 50 days after the onset of treatment. DE genes in S trees may represent a long‐lasting change of the transcriptome that is maintained in the presence of weevils. Previous work highlighted rapidly induced defense responses within hours or days following real or simulated weevil attack (Miller et al., 2005; Ralph et al., 2006, 2008). Features in the rapid early induced response in R trees, such as induced terpenoids and traumatic resin ducts, in addition to differences in constitutive defenses, may contribute to the resistance of R trees.

We used a 2 × 3 factorial design to differentiate insect specific responses. Wounding treatment mimicked the mechanical component of oviposition, although it would not mimic effects of biological elicitors associated with eggs or oviposition fluids (Hilker et al., 2005). This treatment served as a control for mechanical effects of oviposition (Gara & Wood, 1989; Whitehill et al., 2019). The statistical model was parameterized to the experimental design to remove the effect of mechanical wounding from weevil‐specific responses. We identified DE genes as CD between R and S trees or as WI in S trees. One set of genes of particular interest (Set 1) are those that were constitutively expressed at higher levels in R trees. These genes may contribute to effective constitutive defense barriers in R trees against feeding or ovipositing adult weevil or against eggs and developing larvae. Conversely, the lack of or lower expression of such genes may contribute to absence of effective constitutive defense in S trees, which in their geographic location of origin are not co‐evolved with weevils. Set 1 genes were enriched for GO term annotations associated with ‘defense’ compared to ‘growth’. In contrast, genes constitutively higher expressed in S trees (Set 2) were enriched for ‘growth’ GO terms. According to the growth‐differentiation balance hypothesis, well‐defended plants, such as the Sitka spruce R genotype, invest more in constitutive defenses (Herms & Mattson, 1992).

Set 5 comprised another interesting group of genes, which was constitutively expressed at higher levels in R trees and up‐regulated in response to weevil activity in S trees. Almost 40% of the genes in this set are annotated as fungal origin. Reproducible fungal transcriptome signatures may hint towards a role of tree‐ or insect‐associated microbiomes in the spruce‐weevil interaction. Fungal endophytes can contribute to a defensive mutualism against herbivores (Saikkonen et al., 2010). Toxic metabolites produced by endophytic fungi have been implicated in providing protection from spruce budworm in red spruce (*P*. *rubens*; Clark et al., 1989). While fungal transcripts are commonly observed in conifer transcriptomes, associations with spruce weevil interactions have not been previously documented. Weevils are closely related to bark beetles (Shin et al., 2018), which commonly harbor symbiotic fungi (Bracewell & Six, 2015). It is possible that weevil resistance of spruce not only relies on the induced transcriptome response of the plant, but also on gene expression patterns of host‐associated fungi.

## CONFLICT OF INTEREST

The authors declare no conflict of interest.

## AUTHORS CONTRIBUTIONS

J.G.A.W. and J. Bo. planned and designed the research. J.G.A.W performed experiments. J.G.A.W. and M.M.S.Y analyzed data. J.G.A.W. and J. Bo. interpreted the results and wrote the manuscript. All authors reviewed and edited the manuscript.

## Supporting information

Fig S1

Fig S2

Table S1

Table S2

## Data Availability

Transcriptome resources described in this paper are available in NCBI as raw RNA‐Seq under the BioProject Accession PRJNA398042.
