# Supplementary figures and images for: Constitutive and insect‐induced transcriptomes of weevil‐resistant and susceptible Sitka spruce
Source: Plant Environ Interact. 2021 Jun 9;2(3):137–47. doi: 10.1002/pei3.10053 (PMC10168040; doi:10.1002/pei3.10053)

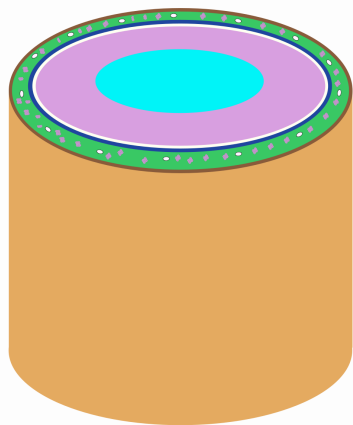

**Control**

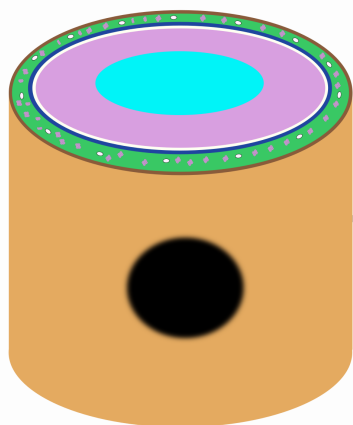

**AOC**

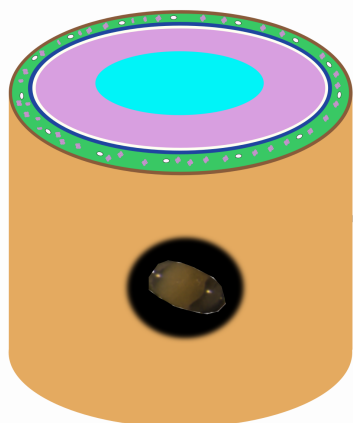

**Egg**

**Experimental Design**

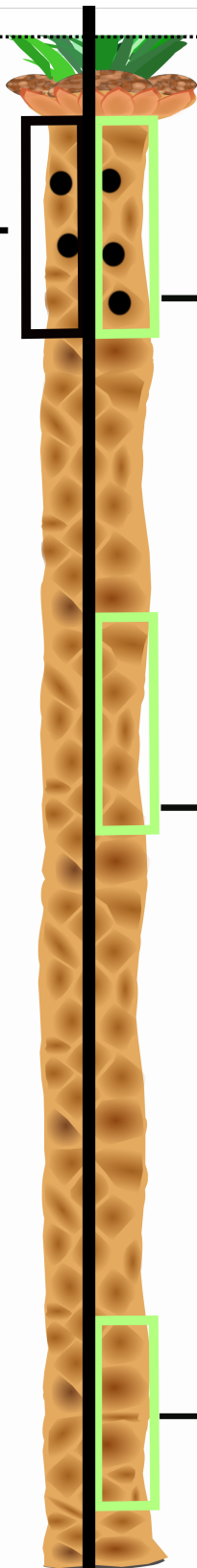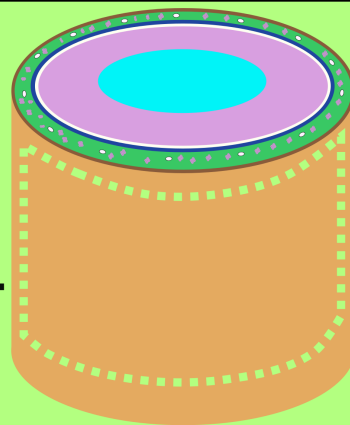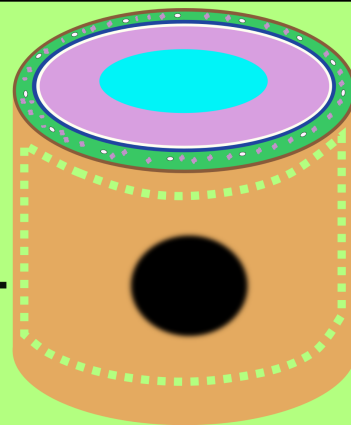

**R Trees**

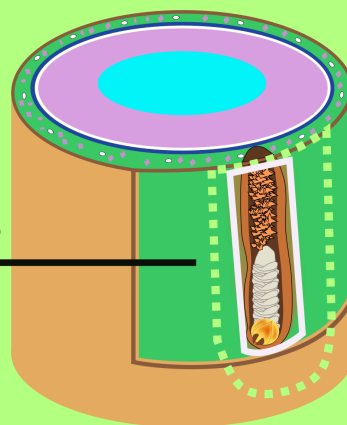

**S Trees**

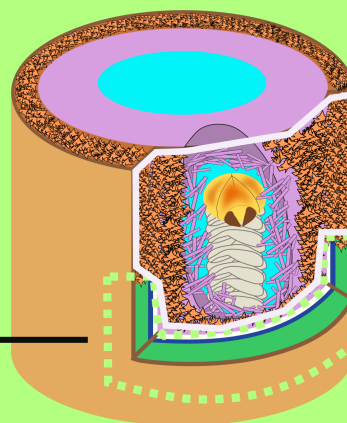

**RNA-seq**

**Sample Collection**

Supplement: Supplementary file 1 — Fig S1 [file PEI3-2-137-s004.pdf]
