# Supplementary material for: Constitutive and insect‐induced transcriptomes of weevil‐resistant and susceptible Sitka spruce
Source: Plant Environ Interact. 2021 Jun 9;2(3):137–47. doi: 10.1002/pei3.10053 (PMC10168040; doi:10.1002/pei3.10053)
Supplement: Supplementary file 2 — Fig S2 [file PEI3-2-137-s001.pdf]

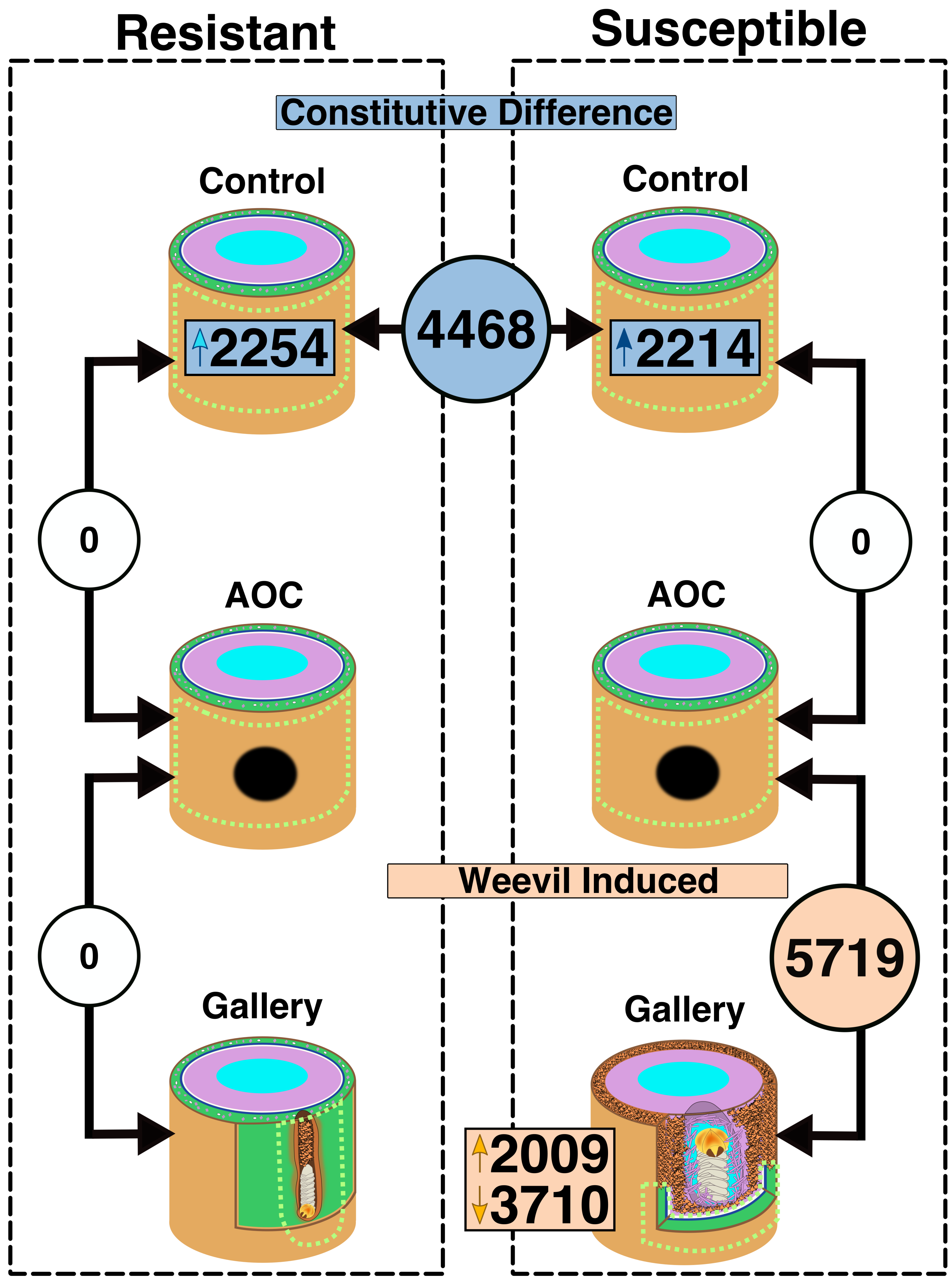
Resistant
Susceptible
Constitutive Difference
Control
Control
4468
2254
2214
0
0
AOC
AOC
Weevil Induced
0
5719
Gallery
Gallery
2009
3710
